# Y-Box Binding Protein-1 Promotes Epithelial-Mesenchymal Transition in Sorafenib-Resistant Hepatocellular Carcinoma Cells

**DOI:** 10.3390/ijms22010224

**Published:** 2020-12-28

**Authors:** Li-Zhu Liao, Chih-Ta Chen, Nien-Chen Li, Liang-Chun Lin, Bo-Shih Huang, Ya-Hui Chang, Lu-Ping Chow

**Affiliations:** Graduate Institute of Biochemistry and Molecular Biology, College of Medicine, National Taiwan University, Taipei 100233, Taiwan; d99442006@ntu.edu.tw (L.-Z.L.); d02442004@ntu.edu.tw (C.-T.C.); nien14lee14@gmail.com (N.-C.L.); koala30135@gmail.com (L.-C.L.); a0938548239@gmail.com (B.-S.H.); yahuichang@ntu.edu.tw (Y.-H.C.)

**Keywords:** hepatocellular carcinoma cell, drug resistance, sorafenib, YB-1, S102 phosphorylation, epithelial-mesenchymal transition

## Abstract

Hepatocellular carcinoma is one of the most common cancer types worldwide. In cases of advanced-stage disease, sorafenib is considered the treatment of choice. However, resistance to sorafenib remains a major obstacle for effective clinical application. Based on integrated phosphoproteomic and The Cancer Genome Atlas (TCGA) data, we identified a transcription factor, Y-box binding protein-1 (YB-1), with elevated phosphorylation of Ser102 in sorafenib-resistant HuH-7^R^ cells. Phosphoinositide-3-kinase (PI3K) and protein kinase B (AKT) were activated by sorafenib, which, in turn, increased the phosphorylation level of YB-1. In functional analyses, knockdown of YB-1 led to decreased cell migration and invasion in vitro. At the molecular level, inhibition of YB-1 induced suppression of zinc-finger protein SNAI1 (Snail), twist-related protein 1 (Twist1), zinc-finger E-box-binding homeobox 1 (Zeb1), matrix metalloproteinase-2 (MMP-2) and vimentin levels, implying a role of YB-1 in the epithelial-mesenchymal transition (EMT) process in HuH-7^R^ cells. Additionally, YB-1 contributes to morphological alterations resulting from F-actin rearrangement through Cdc42 activation. Mutation analyses revealed that phosphorylation at S102 affects the migratory and invasive potential of HuH-7^R^ cells. Our collective findings suggest that sorafenib promotes YB-1 phosphorylation through effect from the EGFR/PI3K/AKT pathway, leading to significant enhancement of hepatocellular carcinoma (HCC) cell metastasis. Elucidation of the specific mechanisms of action of YB-1 may aid in the development of effective strategies to suppress metastasis and overcome resistance.

## 1. Introduction

Hepatocellular carcinoma (HCC) is the most common primary liver malignancy worldwide. The incidence rate of HCC has increased over the year, resulting in 300,000 to 800,000 deaths annually [1,2]. Currently, ~40–50% patients are diagnosed at early stages and considered suitable for curative treatment, while systemic therapy is required for almost half of all patients with advanced-stage tumors. However, limited progress has been made in improving the efficacy of systemic therapy for advanced HCC [3,4].

Sorafenib (Nexavar), a multikinase inhibitor, has been identified as a potent small- molecule inhibitor of Raf kinase, vascular endothelial growth factor receptor, platelet-derived growth factor receptor, Kit receptor tyrosine kinase and Fms-like tyrosine kinase 3, and it is approved for the treatment of advanced HCC by the Food and Drug Administration. In the pivotal Sorafenib Hepatocellular Carcinoma Assessment Randomized Protocol (SHARP) study and the subsequent Asia-Pacific studies, sorafenib improved the median overall survival (OS) by 2–3 months in patients with advanced HCC relative to placebo control [5]. Despite this improvement in survival, the efficacy of sorafenib for HCC is modest, with an objective tumor response rate as low as 2–3% [6]. Furthermore, the majority of patients with HCC eventually develop progressive disease following sorafenib treatment, which presents a major obstacle to successful outcomes. Identification of the critical downstream signaling molecules involved in resistance mechanisms in tumor cells is therefore critical to develop viable options for cancer therapy.

Previous studies suggest that acquired resistance results from factors that develop during sorafenib treatment. Several potential mechanisms of sorafenib resistance have been proposed to date, including epidermal growth factor receptor (EGFR) activation, c-Jun activation, autophagy, protein kinase B (AKT) activation, hypoxic environment, dysregulation of apoptosis, cancer stem cell renewal and epithelial-mesenchymal transition (EMT) [7]. EGFR, AKT and extracellular signal-regulated kinase (ERK) activities have been implicated in both inherent and acquired resistance to sorafenib. For example, elevated EGFR activity is reported to affect the efficacy of sorafenib [8]. Other studies have demonstrated that the phosphoinositide-3-kinase (PI3K)/AKT pathway is critical for induction of resistance to sorafenib and impairment of AKT activity contributes to increased sensitivity to sorafenib of HCC cells [9]. Downregulation of p-ERK is additionally associated with sorafenib resistance of HCC [10,11]. Enhanced metastasis of HCC cells with acquired sorafenib resistance has been documented [12]. Furthermore, a number of studies suggest that EMT is involved in shorter disease-free survival as well as chemoresistance in HCC [13,14]. EMT is critically involved in invasion and metastasis, acquisition and maintenance of stem cell characteristics, resistance to cell death and aging, escape from immune monitoring, and drug resistance of tumor cells [15]. Given the limited availability of promising therapies for progressive HCC, clarification of the downstream signaling pathways related to EMT may be useful to identify significant molecules for therapeutic exploitation.

Previously, we employed a quantitative phosphoproteomic approach to delineate the mechanisms underlying acquired resistance of HCC to sorafenib [16]. By integrating data on significantly upregulated phosphoproteins with genes implicated in poor prognosis in patients with liver cancer from the TCGA database and subjecting the intersecting 148 differentially expressed molecules to biological network analysis, Y-box binding protein-1 (YB-1) was identified as a potential regulatory molecule of this dysregulated downstream network in sorafenib-resistant cells. YB-1 is a multifunctional protein that regulates gene expression at different levels and is reported to activate several genes associated with cancer development and progression. Overexpression of YB-1 in multiple cancers is closely correlated with unfavorable patient prognosis. Accumulating clinical evidences suggests that patients with elevated YB-1 expression are at high risk of relapse and metastasis [17]. The clinical relevance of YB-1 as a gene expression regulator in cancer progression is well documented. Moreover, nuclear localization of YB-1 is associated with drug resistance in cancer. Inhibition of YB-1 expression has been shown to increase the sensitivity of cancer cells to chemotherapy [17,18]. In view of its significant association with tumor progression, invasion, metastasis, and resistance to chemotherapeutic drugs, YB-1 may serve as a biomarkers for cancer progression. Notably, phosphorylation of YB-1 at serine 102 appears necessary for its gene expression regulation activities [18].

In this study, we focused on the molecular mechanisms of action of YB-1 in HCC development and its potential association with sorafenib resistance. The functional significance of YB-1 phosphorylation in tumor malignancy was further investigated.

## 2. Results

### 2.1. Overexpression of YB-1 in HuH-7^R^ Cells Is Associated with Poor Prognosis of Liver Cancer 

**Figure 1:**
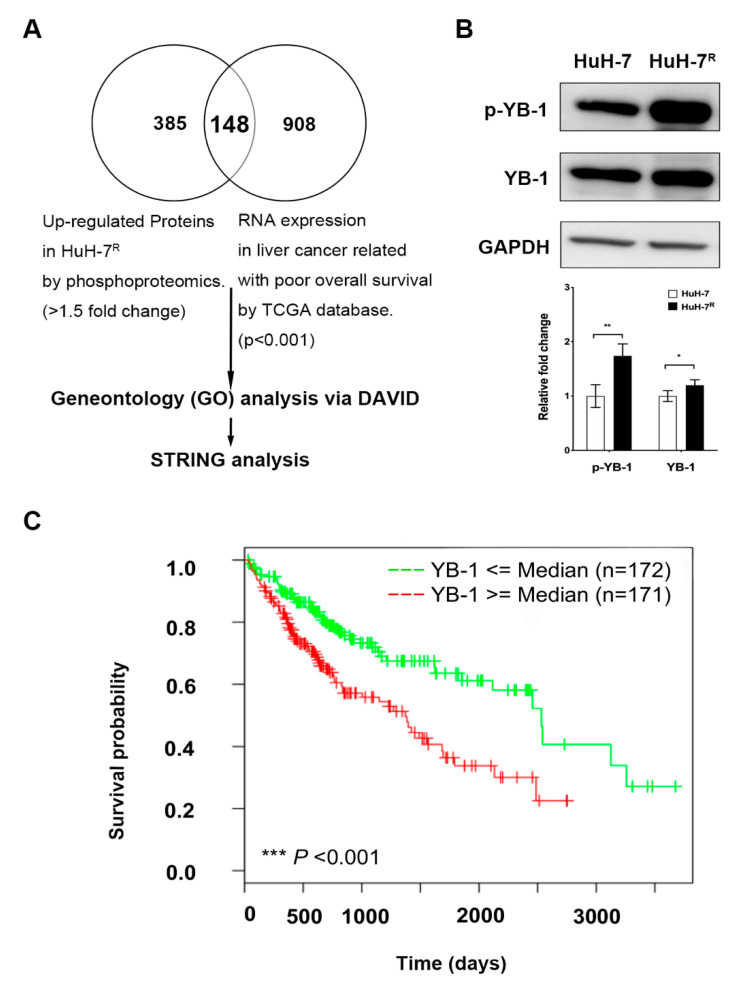
Bioinformatic identification and validation of Y-box binding protein-1 (YB-1) as a potential biomarker in HuH-7^R^ cells. (**A**) Strategy for identification of sorafenib resistance-related differentially expressed molecules in hepatocellular carcinoma (HCC). (**B**) Phosphorylation levels of YB-1 and total YB-1 expression in HuH-7 and HuH-7^R^ cells validated by immunoblotting, upper panel. Relative changes of the expression levels were quantified using GAPDH as normalization control, lower panel. (* *p* < 0.05; ** *p* < 0.01). (**C)** Kaplan-Meir OS curves of the YB-1 gene among 343 HCC patients in the TCGA dataset. The *p* value was calculated with the log-rank test (*** *p* < 0.001).

We performed quantitative phosphoproteomic analyses to analyze phosphoproteins in HuH-7 and HuH-7^R^ cells. The proteins showing >1.5-fold changes (including 533 phosphoproteins) were classified as significantly increased in phosphorylation in resistant HuH-7^R^ cells [16]. To obtain potential candidate genes correlated with poor OS of HCC patients, a total of 1056 genes were identified from the TCGA database (*p* < 0.001). Upon combining the two datasets, we observed overlap of 148 differentially expressed molecules, which were further subjected to gene ontology (GO) analyses using database for annotation, visualization, and integrated discovery (DAVID) (Figure 1A). Functional enrichment analysis revealed the top three significant functions (*p* < 0.05) of the differentially expressed molecules as cytoskeleton (*n* = 40, *p* < 0.001), cell–cell adhesion (*n* = 12, *p* < 0.001) and microtubule-based movement (*n* = 7, *p* < 0.001) (Figure S1). This finding supports the involvement of the differentially expressed molecules in regulation of cytoskeletal structures and the implication of effects of these molecules on cell migration or invasion. Subsequently, the differentially expressed molecules were clustered through Protein-protein interaction (PPI) network construction using STRING. A total of 47 molecules with combination scores ≥ 0.95 were enrolled for PPI network construction. Twenty genes with roles in cellular movement-related functions were identified in the center cluster of the PPI network, and 27 other differentially expressed molecules were determined as potential regulatory genes of those in the center cluster (Figure S2). To further identify significant regulatory factors among the 27 differentially expressed molecules, functional enrichment analysis was performed with GO using DAVID. The top enriched biological process was gene expression (*n* = 11, *p* < 0.001) (Supplemental Table S3). Among the 11 differentially expressed molecules with potential roles in gene expression, transcription was the top enriched function (*n* = 3, *p* value = 0.11) (Supplemental Table S4), including YB-1, pituitary tumor transforming gene 1 (PTTG1), and proliferation-associated protein 2G4 (PA2G4). YB-1 has previously been identified as a metastasis-related molecule [19]. Accordingly, we focused on YB-1 as a potential gene expression regulatory molecule with cellular movement-related functions in drug-resistant HCC. High protein expression and the phosphorylation at Ser102 of YB-1 in HuH-7^R^ cells were validated via immunoblotting (Figure 1B). Compared to HCC patients with low YB-1 expression, those expressing high levels of YB-1 had significantly shorter median OS (3.9 vs. 6.9 years, *p* < 0.001), as determine from analysis of the TCGA dataset (total sample size, *n* = 343) (Figure 1C). Our data suggest that high YB-1 expression is associated with poor OS and may be linked to malignant progression of HCC. Accordingly, we further investigated the role of YB-1 and its potential correlation with sorafenib resistance in HuH-7^R^ cells.

### 2.2. YB-1 Phosphorylation Is Regulated by Different Signaling Pathways Induced by Sorafenib in HuH-7 Cells and HuH-7^R^ Cells

**Figure 2:**
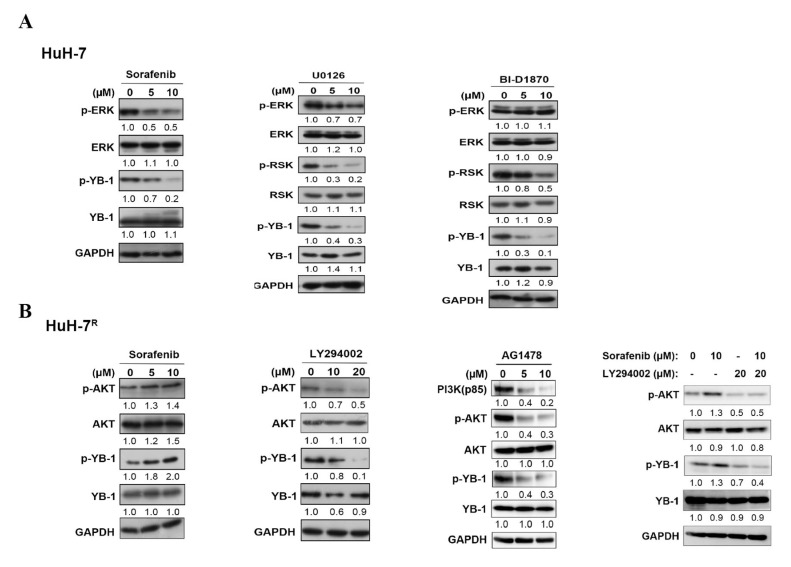
YB-1 phosphorylation is regulated by different signaling pathways in response to sorafenib treatment in HuH-7 and HuH-7^R^ cells. (**A**) HuH-7 cells were incubated with increasing doses of sorafenib, U0126 or BI-D1870 for 12 h. (**B**) HuH-7^R^ cells were incubated with increasing doses of sorafenib, LY294002, AG1478 or sorafenib combined with LY294002 for 12 h. Cell lysates were separated using sodium dodecyl sulfate-polyacrylamide gel electrophoresis (SDS-PAGE) and subjected to immunoblotting with the indicated antibodies. Intensity was quantified using densitometry and normalized to that of GAPDH. Blots are representatives of three separate experiments.

Data from our bioinformatic analyses suggest that YB-1 phosphorylation is involved in facilitating the sorafenib resistance in HuH-7^R^ cells. To characterize the different mechanistic responses of parental HuH-7 and HuH-7^R^ cells to sorafenib treatment, we utilized several inhibitors for the mitogen-activated protein kinase (MAPK) signaling pathway (sorafenib, U0126 and BI-D1870) and EGFR/PI3K/AKT signaling pathway (AG1478 and LY294002). As shown in Figure 2A, upon treatment of HuH-7 cells with sorafenib, phosphorylation levels of ERK1/2 and YB-1 were decreased in a dose-dependent manner, while the phosphorylation level of Akt was not altered (Figure S3A). Inhibition of ERK1/2 and 90 kDa ribosomal protein s6 kinase (RSK) phosphorylation with U0126 (a MEK inhibitor) additionally induced a significant decrease in p-YB-1 level in HuH-7 cells. Phosphorylation levels of RSK and YB-1 were decreased following BI-D1870 treatment in a dose-dependent manner while no changes in the p-ERK1/2 level were observed. Our data clearly suggest that the MAPK pathway is involved in sorafenib-mediated p-YB-1 downregulation in HuH-7 cells. Interestingly, however, we observed a dose-dependent increase in both the phosphorylation of AKT and YB-1 following sorafenib treatment in HuH-7^R^ cells (Figure 2B), even though p-ERK was inhibited at the higher dose (Figure S3B). The effects of AG1478, LY294002 and the combination of sorafenib and LY294002 on the phosphorylation status of AKT and YB-1 in HuH-7^R^ cells were further evaluated. Notably, inhibition of PI3K and AKT phosphorylation with AG1478 or LY294002 treatment led to a significant decrease in YB-1 phosphorylation in HuH-7^R^ cells. In addition, the combination of sorafenib and LY294002 also significantly inhibited in phosphorylation of YB-1 in HuH-7^R^ cells (Figure 2B). Our findings suggest that sorafenib reduces YB-1 phosphorylation through inhibiting the MAPK pathway in HuH-7 cells and conversely enhances YB-1 phosphorylation through activating the PI3K/AKT pathway in HuH-7^R^ cells.

### 2.3. Immunohistochemical Staining of HuH-7 and HuH-7^R^ Tumor-Bearing Mice with and Without Sorafenib Treatment

**Figure 3:**
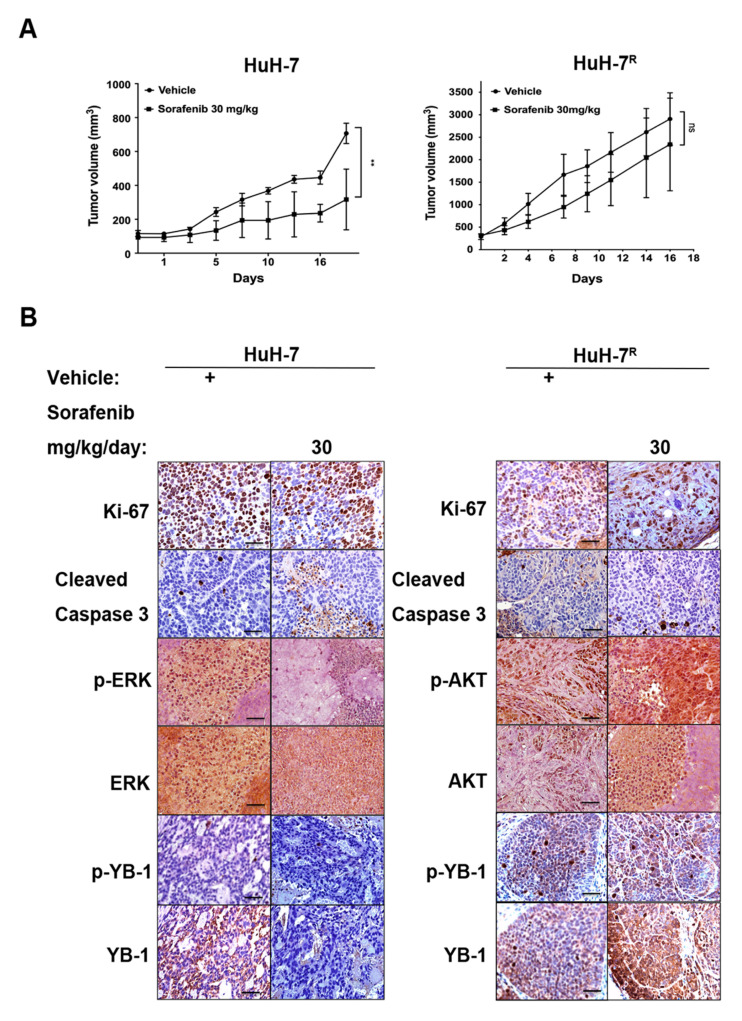
Effects of sorafenib on tumor growth and differentially expressed proteins of HuH-7 and HuH-7^R^ cells in xenograft models. (**A**) In vivo effects of sorafenib on tumor growth of HuH-7 or HuH-7^R^ mice. Nude mice were injected subcutaneously with HuH-7 or HuH-7^R^ cells. Mice were orally administered vehicle or sorafenib 30 mg/kg for 18 days. Tumor volumes at the indicated time-points were calculated and plotted (*n* = 5/group). *p* Values were determined using the *t* test. (ns, not significant; ** *p* < 0.01). (**B**) Changes in specific protein markers in HuH-7 and HuH-7^R^ tumors tissues. Paraffin-embedded sections of tumor tissues were analyzed via immunohistochemistry and stained with the indicated antibodies. Representative sections were captured at 400× magnification.

To further validate the in vivo response of HCC to sorafenib, we employed a mouse xenograft tumor models established by subcutaneous injection of HuH-7 or HuH-7^R^ cells. As shown in Figure 3A, HuH-7 tumor-bearing mice were orally treated with sorafenib (30 mg/kg) or vehicle control for 18 days. Notably, the tumor volumes of sorafenib-treated HuH-7 tumor-bearing mice were significantly suppressed, compared with vehicle controls. In contrast, sorafenib treatment failed to inhibit tumor growth in HuH-7^R^ tumor-bearing mice, resulting in no significant difference to vehicle controls. Immunohistochemical assessment of key marker proteins in the tumor sections from HuH-7-bearing mice revealed a significant reduction in levels of Ki-67 (a proliferation marker), p-YB-1, YB-1, p-ERK1/2 and ERK1/2. Moreover, the level of active cleaved caspase-3, an apoptosis marker, was significantly increased in the presence of sorafenib. Immunohistochemical data revealed strong staining for Ki-67, p-YB-1, YB-1, p-AKT and AKT but not cleaved caspase-3 in mice bearing HuH-7^R^ tumors (Figure 3B). Based on the collective results, we conclude that HuH-7^R^ tumors have greater tumorigenic potential than HuH-7 tumors. Sorafenib inhibited cell proliferation and induces apoptosis in HuH-7 tumors, but it did not have significant effect on HuH-7^R^-derived tumors. Furthermore, sorafenib promoted YB-1 phosphorylation via effects from PI3K/AKT but not MAPK signaling in HuH-7^R^ tumors, which was consistent with our in vitro findings (Figure 2).

### 2.4. YB-1 Knockdown Does Not Affect Proliferation, But Suppresses Migration, Invasion and Sphere Formation in HuH-7^R^ Cells

**Figure 4:**
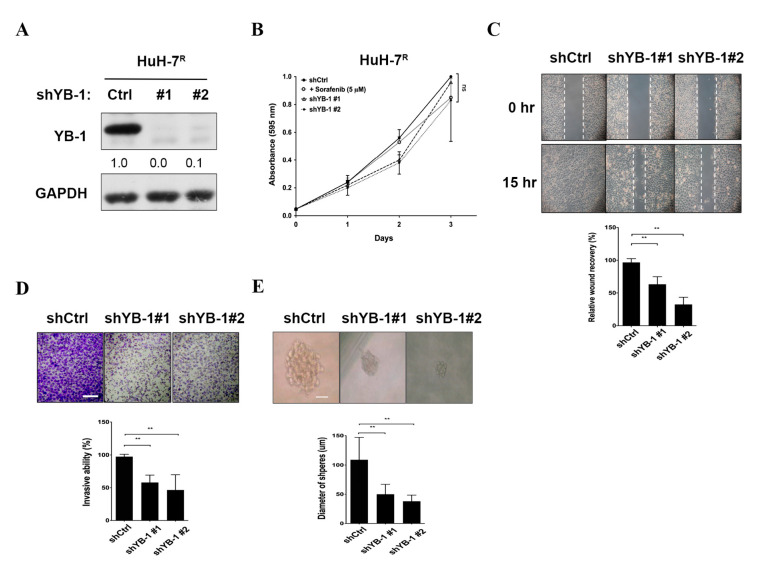
Knockdown of YB-1 does not inhibit proliferation, but suppresses migration, invasion, and sphere formation of HuH-7^R^ cells. (**A**) HuH-7^R^ cells were infected with lentiviruses containing shYB-1 (#1, #2) or shCtrl, and after 48 h, lysed and analyzed for YB-1 expression status by immunoblotting with the indicated antibodies. (**B**) Viability of HuH-7^R^ cells treated with shCtrl, shYB-1 or 5 μM sorafenib determined at the indicated time-points with the MTT ssay. Plots depict cumulative cell absorbance versus days in culture. (**C**) Wound healing assay of YB-1 knockdown HuH-7^R^ cells. The micrographs depict cells migrating into the gap 0 h and 15 h after removal of the insert. (**D**) Transwell invasion assay of YB-1 knockdown HuH-7^R^ cells. Cells in the central field of each insert were visualized using light microscopy and quantified. (**E**) Sphere formation assay of YB-1 knockdown HuH-7^R^ cells. Images of the spheres formed were captured on day 9 and sphere diameters measured. Data were quantified and presented as means ± SD. All results are the representatives of at least three independent biological replicates. Scale bars = 50 µm. *p* Values were determined using the *t* test (ns, not significant; ** *p* < 0.01); shCtrl, control shRNA; shYB-1, shRNA against YB-1.

To establish the potential roles of YB-1 in resistant HCC cell lines, several functional assays were performed. We generated several sorafenib-resistant HCC cell lines (HuH-7^R^, Hep3B^R^, PLC-5^R^, Sk-Hep-1^R^) and compared YB-1expression and Ser102 phosphorylation status with those in parental HCC cell lines via immunoblotting (Figure S4). PLC-5^R^ cells displayed high expression of YB-1 and Ser102 phosphorylation. The sorafenib IC_50_ value of 12.6 μM was slightly higher than that of HuH-7^R^ cells (10.3 μM). Lentiviral-mediated delivery of YB-1 shRNAs (small hairpin RNA) was conducted to inhibit YB-1 expression in HuH-7^R^ and PLC-5^R^ cells. The knockdown efficiency data are presented in Figure 4A and Figure S5A. In the MTT (3-(4,5-dimethylthiazol-2-yl)-2,5-diphenyltetrazolium bromide) assay, proliferation of HuH-7^R^ cells was not markedly different under sorafenib treatment or after YB-1 knockdown, compared with the control group (Figure 4B). Subsequent wound healing and invasion assays performed using YB-1 knockdown and control HuH-7^R^ cells demonstrated that suppression of YB-1 led to a significant decrease in migration (Figure 4C) and invasion activities (Figure 4D), compared with control HuH-7^R^ cells. Furthermore, sphere formation of HuH-7^R^ cells was markedly attenuated upon inhibition of YB-1 (Figure 4E). Similarly, knockdown of YB-1 in PLC-5^R^ cells led to decreased migration, invasion and sphere formation but not cell proliferation (Figure S5B–E). The results clearly demonstrate that YB-1 does not influence cell proliferation but facilitates motility and metastasis of HuH-7^R^ and PLC-5^R^ cells.

### 2.5. YB-1 Enhances Expression of EMT-Related Molecules in HuH-7^R^ Cells

**Figure 5:**
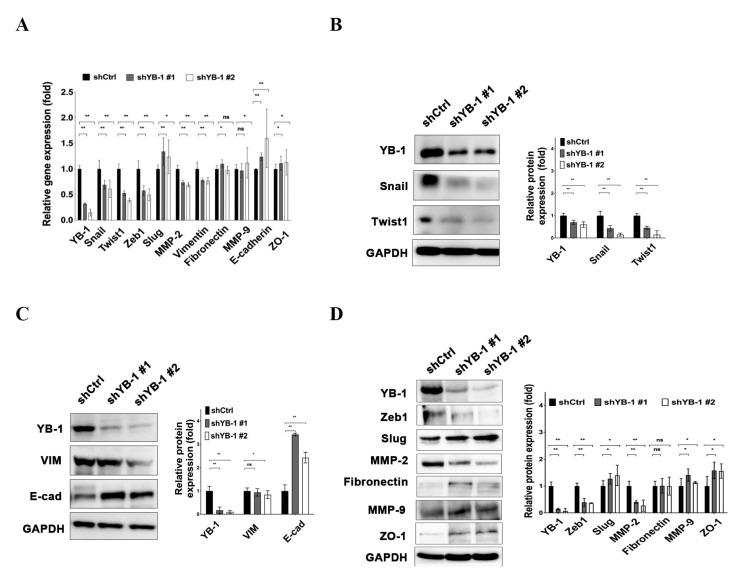
YB-1 enhances epithelial-mesenchymal transition (EMT)-associated gene and protein expression in HuH-7^R^ cells. (**A**) mRNA levels of EMT-associated genes were assessed via quantitative real-time PCR (qPCR) after YB-1 silencing in HuH-7^R^ cells. (**B**–**D**) Protein expression levels of EMT-associated genes after YB-1 knockdown in HuH-7^R^ cells detected by immunoblotting with the indicated antibodies using GAPDH as the loading control. Quantified data are presented as means ± SD. All results are representative of at least three independent biological replicates. shCtrl, control shRNA; shYB-1, shRNA against Y-box binding protein-1. *p* Values were determined using the *t* test (ns, not significant; * *p* < 0.05; ** *p* < 0.01).

To clarify the mechanisms by which YB-1 enhances the migration and invasion of drug-resistant cancer cells, expression levels of the genes and proteins associated with EMT were assessed. Our data showed that mesenchymal genes encoding zinc-finger protein SNAI1 (Snail), twist-related protein 1 (Twist1), zinc-finger E-box-binding homeobox 1 (Zeb1), matrix metalloproteinase-2 (MMP-2)*,* and vimentin (*VIM*) were downregulated and epithelial genes encoding E-cadherin (E-cad) and tight junction protein ZO-1 were upregulated in YB-1-depleted HuH-7^R^ cells. (Figure 5A). Reduced protein expression of Snail, Twist1, Zeb1, MMP-2, and vimentin and the induction of E-cadherin and ZO-1 proteins in YB-1 knockdown HuH-7^R^ cells were determined via immunoblotting (Figure 5B–D). Expression patterns of mesenchymal-related molecules, such as SNAI2 (Slug), MMP-9 and fibronectin, were not significantly different in YB-1 knockdown HuH-7^R^ cells. Our data suggested that the YB-1 is involved in regulation of EMT through modulation of specific genes in HuH-7^R^ cells.

### 2.6. YB-1 Promotes Cdc42-Mediated F-Actin Regulation and Filopodia Formation in HuH-7^R^ Cells

**Figure 6:**
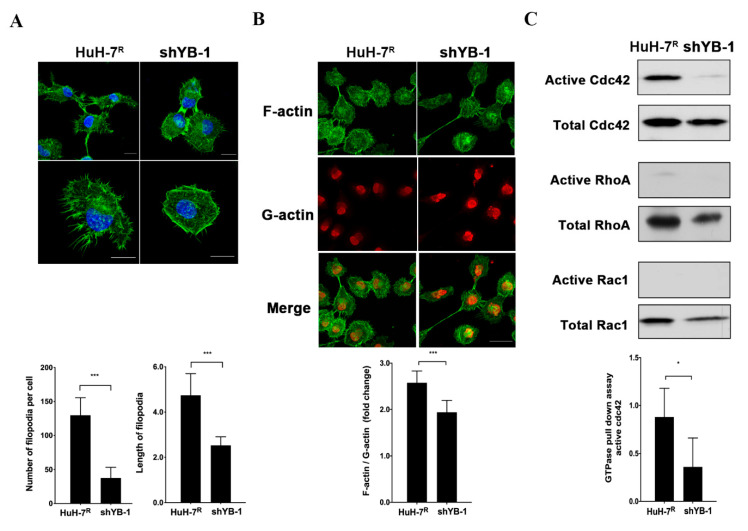
YB-1 regulates filopodia formation through modulating Rho GTPases activity and reorganization of the actin cytoskeleton in HuH-7^R^ cells. (**A**) YB-1 knockdown induced filopodia regression. F-actin was stained with phalloidin-conjugated Alexa Fluor 488 (green). DNA was counterstained with DAPI (blue). The number and length of filopodia per cell were calculated from 20 cells in each group (*** *p* < 0.001, Scale bars = 20 µm). (**B**) G-actin and F-actin were visualized via immunofluorescence staining with Alexa Fluor 488 phalloidin (F-actin, green) and DNase I-conjugated Alexa Fluor 594 (G-actin, red). The F-actin to G-actin ratio per cell was calculated from 50 cells in each group (*** *p* < 0.001, Scale bar = 50 μm). (**C**) Rho-family-GTPase activity was examined using a small GTPase family pull-down assay. Total cell lysates were immunoprecipitated and analyzed by immunoblotting with specific antibodies. Active cdc42 was quantified and normalized to total cdc42. Error bars indicate mean ± SEM, and *p* values were calculated with the *t* test (* *p* < 0.05).

In view of the finding that YB-1 influences cell invasiveness and mobility, the effects of its knockdown on filopodia formation and actin cytoskeleton structure in HuH-7^R^ cells were further assessed. Knockdown of YB-1 caused a significant reduction in the number and length of filopodia formed in HuH-7^R^ cells compared to control cells (Figure 6A). Cdc42, RhoA and Rac1 are members of a Ras-related Rho family of GTP-binding proteins that support a migratory phenotype in cells. Moreover, F-actin formation is an important event in progression of cell motility [20]. Accordingly, we explored the effect of YB-1 inhibition on F-actin structure and GTPase activity of Cdc42, RhoA and Rac1. The F-actin versus G-actin ratio was slightly decreased in YB-1-depletd HuH-7^R^ cells, compared with control cells (Figure 6B). Levels of GTP-bound (active) Cdc42, RhoA and Rac1 were further determined using the GST-pull down assay. The active Cdc42 level was lower in YB-1 knockdown HuH-7^R^ than control cells. However, active RhoA and active Rac1 levels were barely detectable in both knockdown and control cells (Figure 6C). Our collective findings suggest that YB-1 regulates the activity of the GTPase Cdc42, which mediates actin cytoskeleton rearrangement to facilitate filopodia formation and subsequent cell motility. 

### 2.7. YB-1 Ser102 Phosphorylation Is Crucial for Migratory Potential and Invasiveness of HuH-7^R^ Cells

**Figure 7:**
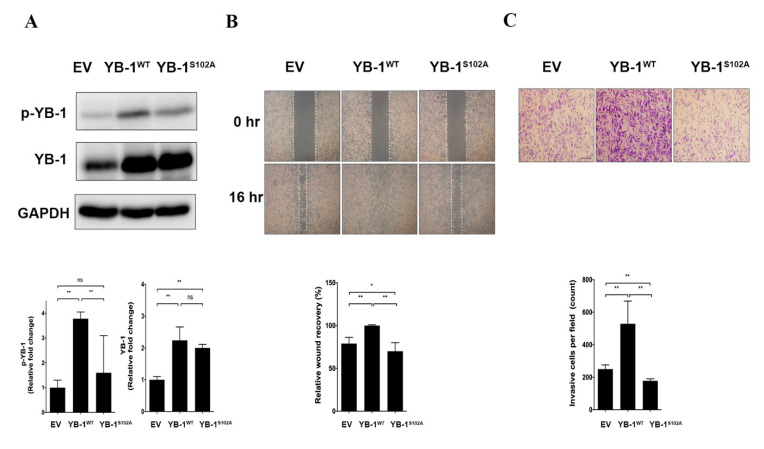
YB-1 S102 phosphorylation is required for migration and invasion of HuH-7^R^ cells. (**A**) HuH-7^R^ cells were infected with lentiviruses containing empty vector control (EV), YB-1 wild-type (YB-1^WT^) or YB-1 serine 102 to alanine mutant (YB-1^S102A^) and after 24 h, lysed and analyzed via immunoblotting with the indicated antibodies, upper panel. The relative expression efficiency of WT and S102A YB-1 was quantified using GAPDH as normalization control, lower panel. (**B**) Wound healing assay of EV, YB-1^WT^ or YB-1^S102A^ -overexpressing HuH-7^R^ cells. The micrographs depict cells that migrated into the gap 0 h and 16 h after removal of the insert, upper panel. Wound recovery was quantified, lower panel. (**C**) Transwell invasion assay of EV, YB-1^WT^ or YB-1^S102A^- overexpressing HuH-7^R^ cells. Cells in the central field of each insert were visualized via light microscopy, upper panel. Invaded cells were quantified, lower panel. *p* values were determined using the *t* test (scale bar = 50 μm; * *p* < 0.05; ** *p* < 0.01).

Our findings suggest that YB-1 is a downstream target of the PI3K/AKT pathway in HuH-7^R^ cells. S102 phosphorylation of YB-1 was reported to enhance tumor cell migration and invasion [21]. To clarify the mechanisms underlying enhanced tumorigenicity elicited by p-YB-1, mutational assays were performed on HuH-7^R^ cells. To this end, a YB-1 S102A mutant was constructed in a lentivirus expression-based vector. Overexpression of empty vector control (EV), YB-1 wild-type (YB-1^WT^) or YB-1 S102A mutant (YB-1^S102A^) in HuH-7^R^ cells, was accomplished using the lentivirus-mediated delivery method. The expression efficiency is presented and quantified in Figure 7A. Wound healing (Figure 7B) and transwell (Figure 7C) assays revealed decreased motility and invasiveness of HuH-7^R^ cells following the inhibition of YB-1 S102 phosphorylation, compared to the control cells. The collective results indicate that S102 phosphorylation of YB-1 is mediated by AKT affects the migration and invasiveness of HuH-7^R^ cells.

## 3. Discussion

Sorafenib is a kinase-targeting drug commonly used for treatment of advanced HCC, but the majority of patients eventually develop drug resistance. To enhance therapeutic efficacy, elucidation of the molecular changes related to sorafenib resistance is critical. Previous studies have described the involvement of various aberrant signaling pathways in progression of HCC. Primary resistance to sorafenib is associated with sustained activation of EGFR downstream signaling molecules, including Raf/Ras/MEK/ERK, overexpression of JNK, and abnormal activation of VEGFA [10]. Additionally, PI3K/AKT and JAK-STAT pathways are reported to be involved in acquired resistance to sorafenib of HCC [10,22]. Elucidation of the key molecules in aberrant pathways implicated in the development of sorafenib resistance may thus facilitate patient stratification for optimization of therapeutic outcomes. 

To screen protein candidates potentially activated by sorafenib, we identified the key molecules involved in aberrant signaling pathways in sorafenib-resistant HuH-7^R^ cells. In this study, 148 differentially expressed molecules were selected from intersective combination of MS analysis and TCGA datasets, and PPI network construction was employed to identify the key molecules in sorafenib-resistant HCC cells. A total of 20 differentially expressed molecules were mainly linked to cellular movement-related functions and shown to be critical for tumor progression [23,24,25]. NOP56 and ANLN are E-cadherin binding proteins, which might be associated with E-cadherin-mediated cell-cell adhesion in epithelial tissues [26]. TUBA1B, TUBA1C, TUBB2A and TUBB4B belong to the tubulin family and form the microtubule filaments, which usually play important roles in mesenchymal migration [27,28]. NEDD1 and CKAP5 are also involved in microtubule system by promoting the nucleation of microtubules, whereas STMN1 regulates microtubule filament by destabilizing microtubules [29]. DYNC1H1, a dynein motor protein, and KIF18B, KIF23 and KIF4A, members of kinesin superfamily motor proteins, assist cargo transport along microtubules to various subcellular locations, such as the leading edge of migrating cells [30]. ALDOA and RACGAP1 are tubulin binding proteins important for the microtubule filaments system, and are mainly responsible for metastasis and invasiveness in various carcinomas [31,32]. Not only the microtubule system, some of the identified upregulated molecules are involved in cellular metastasis and carcinogenesis through dysregulating cell division, including cell cycle regulators, CDCA8, CDK1, CDK2, CEP55 and CHEK1 [33,34,35,36,37,38]. Moreover, these 20 proteins were modulated by 27 cellular movement-related regulators. Several of these regulators are responsible for genomic integrity. TOP2A, PTTG1 and SMC4 play central roles in chromosome stability and ERCC6 takes part in excision repair, of which dysregulation drives deleterious chromosomal aberrations and thus carcinogenesis [39,40,41,42]. Moreover, it has been reported that regulation of mRNA stability can govern cancer metastasis [43]. FTSJ3 and DKC1are in charge of RNA modification; DDX10, MKI67and NOP58 are RNA-binding proteins; and NUP188 plays a role in mRNA transport. Dysregulation of these molecules may lead to malignancy [44,45,46,47,48,49]. Dysregulation of protein metabolism is another known mechanism for promoting oncogenesis [50]. EIF2S2 is an anabolic factor, while PSMD1 is a catabolic factor, both of which are correlated to poor cancer prognosis [51,52]. Cell cycle regulators CDC23 and BUB1 have been regarded as EMT-related regulatory proteins [53,54]. Besides, dynamic changes in alternative splicing occur during EMT. hnRNPM is a splicing regulator that promotes an epithelial splicing program [55]. Lastly, PA2G4, PTTG1, KPNA2 and YB-1 are gene expression regulators, of which overexpression usually cause poor clinical outcomes in various malignancies [56,57,58,59]. In the enrichment analysis, the top-ranked molecules were PA2G4, PTTG1 and YB-1, which may act as transcription factors. PA2G4, also designated ErbB3 receptor binding protein 1 (EBP1), is a well-conserved DNA/RNA binding protein that modulates transcriptional activity in different cancer cell types [60,61]. Patients with high expression levels have poor clinical outcomes [62]. PTTG1, an oncogenic transcription factor, is overexpressed in various malignancies and associated with enhanced cell migration and invasion [63]. YB-1, another important gene expression regulator, is an unfavorable prognostic marker in primary HCC and associated with advanced stages of HCC [19]. The multifunctional YB-1 protein promotes expression of genes involved in cell proliferation, migration and invasion of HCC [64]. Our findings suggest that the three cancer-associated gene expression regulators, PA2G4, PTTG1 and YB-1, play important roles in progression of HCC. YB-1 has not been previously characterized as a mediator of sorafenib resistance. Data from phosphoproteomic analyses indicate that upregulation of YB-1 phosphorylation could serve as a mechanism to facilitate sorafenib resistance in HuH-7^R^ cells. Based on correlation analysis with TCGA data, high YB-1 levels were associated with shorter median OS in patients with HCC, supporting a critical role in sorafenib resistance of HCC.

Several aberrant signaling pathways are implicated in acquisition of HCC resistance to sorafenib [10]. For instance, the PI3K/AKT pathway contributes to sorafenib resistance via crosstalk with the MAPK/ERK pathway [65]. Earlier studies have demonstrated that sorafenib activates the PI3K/AKT pathway and that blockage of this pathway enhances the efficacy of sorafenib [66,67]. The PI3K/AKT pathway is involved in development and progression of HCC and shown to be activated in 93% HCC specimens [68,69]. Another study similarly reported activation of AKT in sorafenib-resistant HCC cells, consistent with these findings [70]. In the current investigation, protein expression and phosphorylation levels of Ser 102 of YB-1 were higher in HuH-7^R^ than parental HuH-7 cells. Notably, phosphorylation of YB-1 at Ser102 is reported to be activated by both MAPK/ERK and PI3K/AKT signaling pathways in different cancer cells [71,72]. To establish the YB-1 phosphorylation responses in parental HuH-7 and HuH-7^R^ cells after sorafenib treatment, various inhibitors were employed. Our results showed that p-YB-1 was significantly suppressed by sorafenib, U0126 and BI-D1870, indicating that regulation of YB-1 by the MAPK pathway in HuH-7 cells. In addition, p-YB-1 was significantly increased by sorafenib but decreased by LY294002 and AG1478 in HuH-7^R^ cells, supporting a role as a downstream effector of the EGFR/PI3K/AKT pathway. Experiments with xenograft tumor models disclosed increased levels of p-YB-1 and p-AKT in HuH-7^R^ cells, compared to HuH-7 cells. The results further confirm that sorafenib enhances YB-1 phosphorylation via effects from PI3K/AKT but not MAPK signaling in HuH-7^R^ tumors in vivo. Moreover, elevated p-YB-1 expression was associated with a higher rate of tumor progression. Taken together, these findings suggest that the levels of p-YB-1 regulated by PI3K/AKT signaling mediate progression of HCC and resistance to sorafenib.

Previously, we showed that long-term exposure of HuH-7 cells to sorafenib led to an alteration in morphology into spindle-shaped cells [70]. Sorafenib-resistant cells displayed activation of the EMT process with enhanced invasive and metastatic potential [7]. In our experiments, inhibition of YB-1 functions impaired tumor migration, invasion and sphere formation in HuH-7^R^ and PLC-5^R^ cell lines. To further investigate if the elevated YB-1 expression and phosphorylation after sorafenib treatment is universal in HCC cells, PLC-5, Sk-Hep-1, and Hep3B were subjected to sorafenib training. Among the three tested HCC cell lines, only PLC-5^R^ showed strong expression of YB-1 with simultaneously upregulated Ser102 phosphorylation after sorafenib training. In contrast, Hep3B^R^ and Sk-Hep-1^R^ cells did not display significant increase in either YB-1 or its phosphorylation levels. Heterogeneous composition of tumor cells has been proposed and observed in many cancer types, including liver cancer and non-small cell lung cancer (NSCLC) [73,74]. Drug resistance-induced tumor recurrence has been attributed in parts to heterogeneous tumor cell subpopulations, such as cancer stem cells [74]. Therefore, it is suggested that drug-resistant cancer cells, such as HuH-7^R^ and/or PLC-5^R^ cells, may share a certain similar characteristic of a heterogeneous liver tumor. On the other hand, the original nature and responses to sorafenib treatment of Hep3B and Sk-Hep-1 may be undergone by different mechanisms. Moreover, the YB-1 S102A mutation impaired migration and invasiveness of HuH-7^R^ cells, indicating that phosphorylation of S102 is critical for the modulation of a number of YB-1-induced EMT genes.

To elucidate the potential molecular mechanisms governing the actions of YB-1, we analyzed the expression levels of genes and proteins associated with EMT in YB-1 knockdown cells. Our data revealed downregulation of the EMT transcription factors Zeb1, Twist1, and Snail after YB-1 knockdown in HuH-7^R^ cells. Zeb1 belongs to the zinc family of transcription factors, which suppress the expression of cell polarity factors and activate matrix metalloproteinases (MMP), thus promoting remodeling of the basement membrane and invasion of tumor cells to the surrounding tissue [75]. YB-1 acts as a gene expression regulator at various levels and regulates the corresponding genes through splicing, mRNA stability, translation, or transcription [76]. YB-1 was reported to be recruited by lncRNA-BX111887 at the promoter region of Zeb1, upregulated Zeb1 transcription, and promoted cell invasion and metastasis in pancreatic cancer [77]. Snail, an EMT-inducing transcription factor, plays an important role in cancer invasion. Expression of Snail is correlated with metastasis and poor prognosis in HCC [78,79]. Previous reports have shown that knockdown of YB-1 suppresses expression of Snail in human malignancies [64,80]. We additionally observed that YB-1 regulates expression of Snail, suggesting that YB-1 enhances Snail activity to promote mesenchymal changes in HuH-7^R^ cells. Twist1, another transcription factor, belongs to the basic helix-loop-helix family of proteins. The protein promotes migration and invasion, induces cancer stem cell-like phenotype and mediates chemoresistance [81]. Previous studies have demonstrated that YB-1 serves a main target of Twist [82]. Interestingly, our results showed downregulation of Twist1 at both mRNA and protein levels upon silencing of YB-1 in HuH-7^R^ cells. Earlier microarray data further suggest that YB-1 enhances Twist1 expression at both the mRNA and protein levels [83]. Here, Twist is proposed to be a downstream target of YB-1 in HuH-7^R^ cells. However, the mechanisms underlying the regulation of Twist by YB-1 require further investigation.

Additionally, in our experiments, YB-1 knockdown reduced expression of the mesenchymal markers, MMP-2 and vimentin, and increased expression of the epithelial markers, E-cadherin and ZO-1, in HuH-7^R^ cells. MMP-2, a member of the MMP family, is a proteolytic enzyme that degrades the extracellular matrix to promote invasiveness. Expression and activity of MMP-2 are correlated with poor prognosis, invasion, and metastasis in many human cancer types [84]. YB-1 is reported to regulate MMP-2 expression through interacting at the promoter region of MMP [85]. Vimentin plays a role in maintaining cell shape, stabilizing cytoskeletal interactions, and cell motility [86]. Earlier, YB-1 was shown to enhance vimentin expression and drive tumor progression via EMT in colorectal cancer [87]. However, the mechanisms by which YB-1 enhances vimentin expression in HuH-7^R^ cells are unclear at present. 

E-cadherin mediates cell–cell adhesion, and loss of its expression results in increased motility and invasiveness [79]. Snail is reported to block E-cadherin transcription through binding to specific DNA sequences [88]. Our experiments showed that YB-1 knockdown reduces Snail expression, suggesting that YB-1 promotes activation of Snail and reduction of E-cadherin to facilitate cell migration ability. ZO-1 belongs to the tight junction protein family and plays important roles in maintaining cell–cell adhesion [89]. Further studies are necessary to determine the mechanisms by which YB-1 induces suppression of E-cadherin and ZO-1 expression.

During EMT, numerous cytoskeletal proteins are altered, leading to cytoskeleton rearrangement [90]. The Rho family of GTPases (Cdc42, Rac1 and Rho) play essential roles in the dynamic changes of actin and formation of filopodia during cell migration [20]. Data from the present study showed that YB-1 promotes Cdc42 GTPase activity, which mediates F-actin formation to facilitate filopodia generation in HuH-7^R^ cells but does not activates Rac1 or Rho. Based on the collective results, it is proposed that YB-1 modulates specific EMT-related molecules and stimulates the GTPase family protein, Cdc42, to promote cell migration and invasion.

In summary, YB-1 promotes invasion and migration through the EGFR/PI3K/AKT signaling pathway in sorafenib-resistant HCC. Our findings support the utility of YB-1 as a useful prognostic biomarker and potential molecular target for overcoming sorafenib resistance in HCC.

## 4. Materials and Methods

### 4.1. Cell Culture, YB-1 Knockdown, and Mutant YB-1 Expression

The HCC cell line, HuH-7, was obtained from the Health Science Research Resources Bank (JCRB0403). Hep3B, PLC-5, Sk-Hep-1, and HEK293T cells were acquired from the American Type Culture Collection. Sorafenib-resistant HCC cell lines (HuH-7^R^, Hep3B^R^, PLC-5^R^, and Sk-Hep-1^R^) were established by long-term exposure of each cell type to sorafenib as described previously [91]. HuH-7, HuH-7^R^, Hep3B, Hep3B^R^, PLC-5, PLC-5^R^, Sk-Hep-1, Sk-Hep-1^R^, and HEK293T cells were cultured in Dulbecco’s modified Eagle’s medium (DMEM; HyClone) containing 10% fetal bovine serum, penicillin (100 U/L), and streptomycin (10 mg/L) in a 5% CO_2_ incubator at 37 °C. Lentivirus was produced as described previously [70]. Two target sequences for YB-1 knockdown are: CCAGTTCAAGGCAGTAAATAT (shYB-1#1) and AGCAGACCGTAACCATTATAG (shYB-1 #2). Vectors expressing small hairpin RNAs (shRNA) targeting YB-1 (shYB-1 #1 and #2) or control shRNA (shCtrl) were co-transfected with packaging plasmids in HEK293T cells, after which lentiviruses were harvested and further used to infect HuH-7^R^ or PLC-5^R^ cells. After selection with 1.25 μg/mL puromycin in DMEM for three days, stable clones were used for subsequent assays. For generation of mutant YB-1, the gene was cloned from a cDNA library generated from HuH-7^R^ cells. Substitution of the putative phosphorylation site to alanine was performed using a transformer site-directed mutagenesis by PCR as described previously [92]. Following replacement of Ser102 of YB-1 with alanine, the gene was cloned into the lentivirus construct for expression. Functional assays were performed as described below after confirmation of expression via immunoblotting.

### 4.2. Bioinformatic Analysis 

In an earlier study, we have performed quantitative phosphoproteomic analyses to compare the proteomes of HuH-7 and HuH-7^R^ cells [16]. Phosphoproteins displaying >1.5-fold changes were considered significantly increased in phosphorylation in HuH-7^R^ cells. Overexpressed genes showing a significant correlation with poor OS in HCC patients were obtained from The Cancer Genome Atlas database (TCGA, https://cancergenome.nih.gov/) and intersected with significantly upregulated phosphoproteins to obtain the differentially expressed molecules. Functional enrichment analyses of these differentially expressed molecules were subsequently performed using gene ontology (GO) with the database for annotation, visualization, and integrated discovery (DAVID) tool. The ranking and significance of biological functions were evaluated based on *p* values. Protein–protein interaction (PPI) networks of the differentially expressed molecules were illustrated with STRING, where a combined reliability combined score >0.4 was taken as a significant interaction pair.

### 4.3. Immunoblotting, Immunohistochemistry (IHC) and Immunofluorescent Staining

Protein was extracted from the cells using lysis buffer containing 50 mM Tris-HCl, 150 mM NaCl, 1 mM EDTA, 0.1% SDS, 1% Nonidet P-40, 0.5% sodium deoxycholate, 1× phosphatase inhibitor, and 1× protease inhibitor cocktails, pH 7.4. Cell lysates were separated using sodium dodecyl sulfate-polyacrylamide gel electrophoresis (SDS-PAGE) and transferred onto polyvinylidene fluoride membranes (Millipore). After blocking 5% skim milk in buffer containing 20 mM Tris-HCl and 150 mM NaCl, membranes were incubated in 5% skim milk containing the indicated primary antibodies. A total of 25 commercial antibodies were used for immunoblotting (Supplemental Table S1). For immunohistochemical experiments, sample slides prepared from 5 μm sections of paraffin-embedded xenograft tumors harvested from in vivo animal experiments were deparaffinized, and expression of p-YB-1 S102, YB-1, cleaved caspase-3, Ki-67, p-ERK, ERK, p-AKT, and AKT was examined. Immunoblotting and immunohistochemistry analyses were conducted as described previously [16]. For F-actin analysis, shCtrl and shYB-1 containing HuH-7^R^ cells were stained with Alexa 488-conjugated phalloidin (Thermo Fisher, Waltham, MA, USA), and for G-actin staining, Alexa 594-conjugated deoxyribonuclease I (Thermo Fisher, Waltham, MA, USA) was used in accordance with the manufacturer’s instructions. Cell nuclei were counterstained with 4′,6-diamidino-2-phenylindole (DAPI). Images were captured using a Carl Zeiss LSM 880 confocal microscope (ZEISS, Jena, Germany).

### 4.4. Cell Proliferation, Viability, Wound Healing, Invasion, and Sphere Formation Assays 

Cell proliferation and viability were evaluated using the 3-(4,5-dimethylthiazol-2-yl)-2,5-diphenyltetrazolium bromide (MTT) assay (Sigma–Aldrich, St. Louis, MO, USA), and the IC_50_ values were determined by measuring absorbance at 570 nm. Cell migration ability was assessed with a scratch wound healing assay and the invasive capability of cells was determined using a Matrigel-coated Boyden chamber assay as described previously [93]. The sphere formation assay was performed for 9 days using an ultra-low attachment plate (Corning Inc., New York, NY, USA), and the sphere formation capability was evaluated as described previously [94]. 

### 4.5. Quantitative Real-Time PCR (qPCR), Chromatin Immunoprecipitation (ChIP), and Pull-Down Assays

RNA was extracted using the TRIzol Reagent (Invitrogen, Waltham, MA, USA) following the protocol provided by the manufacturer. Expression of mRNAs was quantified via qPCR using GAPDH as an internal control for normalization. The qPCR reaction was performed using a SensiFAST SYBR Hi-ROX kit (Bioline, London, UK). For the ChIP assay, cells were incubated with specific antibodies, cross-linked, and quenched [70]. Subsequently, cells were lysed and sonicated to yield 200–1000 bp DNA fragments. All ChIP assays were performed with the SimpleChIP Enzymatic Chromatin IP Kit (Cell Signaling Technology, Danvers, MA, USA). The specific primers used for qPCR are presented in Supplemental Table S2. A GST pull-down assay using active GTP-bound Rho A, Rac 1, and Cdc42 was performed using the Cytoskeleton assay kit (RhoA/Rac1/Cdc42 activation assay Combo Biochem Kit; Cytoskeleton, Inc., Denver, MA, USA). Briefly, equal amounts of cell lysates were incubated with Rhotekin-RBD or GST-PBD beads to pull down Rho A, Rac 1, and Cdc42. Next, proteins on the beads and input control lysates were subjected to electrophoresis and detected via immunoblotting. Rho A, Rac 1, and Cdc42 activity was normalized to the total amounts of proteins in cell lysates separately in each sample.

### 4.6. Subcutaneous Xenograft Tumor Models 

Mouse subcutaneous tumor xenografts were established as described previously [70]. When tumor sizes reached 100 mm^3^, dimensions were measured with a caliper at 2-day intervals, Tumor weights were measured with a weighting scale device. Tumor volumes were determined using the formulated: 1/2[(width)^2^ × length] (in cm^3^). All animal studies followed the guidelines of the Institutional Laboratory Animal Care and Use Committee of National Taiwan University. All animal experiments and animal care were performed according to institutional guidelines at the Laboratory Animal Center of National Taiwan University College of Medicine and approved by the National Taiwan University College of Medicine and College of Public Health Institutional Animal Care and Use Committee (IACUC) (IACUC Approval No: 20180355).

### 4.7. Statistical Analysis 

Statistical analyses were performed using GraphPad Prism 7 software (GraphPad, San Diego, CA, USA). Data were presented as means ± SD from three independent experiments. All tests were two-tailed, and *p* values set as * *p* < 0.05, ** *p* < 0.01, or *** *p* < 0.001 for designation of statistical significance.

## Data Availability

The data presented in this study are available on request from the corresponding author.

## Supplementary Materials

The following are available online at https://www.mdpi.com/1422-0067/22/1/224/s1.

## Abbreviations

YB-1	Y-box binding protein-1
HCC	Hepatocellular carcinoma
EMT	epithelial-mesenchymal transition
PTTG1	pituitary tumor transforming gene 1
PA2G4	proliferation-associated protein 2G4
MAPK	mitogen-activated protein kinase
RSK	ribosomal protein s6 kinase
EGFR	epidermal growth factor receptor
PI3K	phosphoinositide-3-kinase
AKT	protein kinase B
Snail	zinc-finger protein SNAI1
Slug	zinc-finger protein SNAI2
Twist1	twist-related protein 1
Zeb1	zinc-finger E-box-binding homeobox 1
MMP-2	matrix metalloproteinase-2
MMP-9	matrix metalloproteinase-9
VIM	vimentin
