# Supplementary material for: Y-Box Binding Protein-1 Promotes Epithelial-Mesenchymal Transition in Sorafenib-Resistant Hepatocellular Carcinoma Cells
Source: Int J Mol Sci. 2020 Dec 28;22(1):224. doi: 10.3390/ijms22010224 (PMC7795419; doi:10.3390/ijms22010224)
Supplement: Supplementary file 1 [file ijms-22-00224-s001.pdf]

Supporting information

Y-box binding protein-1 promotes epithelial-mesenchymal transition in sorafenib-resistant hepatocellular carcinoma cells

Li-Zhu Liao[1], Chih-Ta Chen[1], Nien-Chen Li[1], Liang-Chun Lin [1], Bo-Shih Huang[1], Ya-Hui Chang[1], Lu-Ping Chow[1]

[1]Graduate Institute of Biochemistry and Molecular Biology, College of Medicine, National Taiwan University, Taipei, Taiwan

* Correspondence to chowip@ntu.edu.tw

Supplemental Table 1. List of antibodies used in this study

**Antibodies**	**Providers**
p-YB-1, p-ERK, ERK, p-RSK, RSK, p-AKT, cleaved caspase-3	Cell Signaling Technology
YB-1, AKT, GAPDH, PI3K (p85)	Santa Cruz Biotechnology, Santa Cruz, CA
Snail, Twist1, Zeb1, Slug, MMP-2/-9, Fibronectin, Vimentin, E-cadherin, ZO-1	GeneTex, Irvine, CA
Cdc42, RhoA, Rac1	Cytoskeleton, Inc (CO), USA
Ki-67	Dako Agilent, US and CA

Supplemental Table 2. Primer sequences of target mRNAs for qPCR

Gene	Method	Direction	Primers
GAPDH	qPCR	Forward	GGTCGGAGTCAACGGATTT
		Reverse	TGGACTCCACGACGTACTCA
YB-1	qPCR	Forward	CGCAGTGTAGGAGATGGAGAG
		Reverse	GAACACCACCAGGACCTGTAA
Snail	qPCR	Forward	CCCCAATCGGAAGCCTAACT
		Reverse	CGTAGGGCTGCTGGAAGGTA
Twist1	qPCR	Forward	GGAGTCCGCAGTCTTACGAG
		Reverse	TCTGGAGGACCTGGTAGAGG
Zeb1	qPCR	Forward	GCACCTGAAGAGGACCAGAG
		Reverse	TGCATCTGGTGTTCCATTTT
Slug	qPCR	Forward	GAGCATTTGCAGACAGGTCA
		Reverse	TCCTCATGTTTGTGCAGGAG
MMP-2	qPCR	Forward	GATACCCCTTTGACGGTAAGGA
		Reverse	CCTTCTCCCAAGGTCCATAGC
Vimentin	qPCR	Forward	AGTCCACTGAGTACCGGAGAC
		Reverse	CATTTCACGCATCTGGCGTTC
Fibronectin	qPCR	Forward	CGGTGGCTGTCAGTCAAAG
		Reverse	AAACCTCGGCTTCCTCCATAA
MMP-9	qPCR	Forward	TTGGTCCACCTGGTTCAACT
		Reverse	ACGACGTCTTCCAGTACCGA
E-cadherin	qPCR	Forward	ATTTTTCCCTCGACACCCGAT
		Reverse	TCCCAGGCGTAGACCAAGA
ZO-1	qPCR	Forward	CGAGTTGCAATGGTTAACGGA
		Reverse	TCAGGATCAGGACGACTTACTGG

Supplemental Table 3. The top six enriched biological processes for 27 potential regulatory genes of cellular movement-related differentially expressed molecules

**Biological process**	**Molecules**	**# Molecules**	***p* value**
Gene expression	DDX10, DKC1, EIF2S2, ERCC6, FTSJ3, HNRNPM, NOP58, NUP188, PA2G4, PTTG1, YB-1	11	2.33E-05
Nucleic acid metabolic process	DDX10, DKC1, ERCC6, FTSJ3, HNRNPM, KPNA2, NOP58, PA2G4, PTTG1, TOP2A, YB-1	11	7.20E-05
RNA processing	DDX10, DKC1, FTSJ3, HNRNPM, NOP58, PA2G4, YB-1	7	9.84E-05
Regulation of RNA stability	DKC1, HNRNPM, PSMD1, YB-1	4	1.09E-04
Regulation of nuclear cell division	BUB1, CDC23, MKI67, PTTG1	4	1.21E-04
Meiotic cell cycle	BUB1, PTTG1, SMC4, TOP2A	4	2.00E-04

Supplemental Table 4. The top three enriched molecular functions for gene expression-related differentially expressed molecules

**Molecular function**	**Molecules**	**# Molecules**	***p* value**
Transcription factor activity	PA2G4, PTTG1, YB-1	3	0.11
DNA binding	ERCC6, PA2G4, YB-1	3	0.26
Alternative splicing	DKC1, NPM, NUP188, PA2G4	4	0.95

Supplemental Figure legends

Figure S1. Functional enrichment analysis of 148 differentially expressed molecules.

Enriched functions of 148 differentially expressed molecules analyzed using GO with DAVID further ranked based on $-\log_{10} p$ value

Figure S2. PPI network of 47 differentially expressed molecules associated with cellular-related movement functions.

A total of 47 differentially expressed molecules with combination scores greater than 0.95 were enrolled into PPI network via STRING analysis. Twenty cellular movement-related differentially expressed molecules are shown in the cluster (marked as a blue dashed line) and 27 differentially expressed molecules around the cluster identified as regulatory genes of the cellular movement-related molecules.

Figure S3. HuH-7 and HuH-7^{R} cells responded differently toward sorafenib treatment upon YB-1 regulation. (A) HuH-7 cells were incubated with increasing doses of sorafenib for 12 h. The changes of p-Akt and p-YB-1 were monitored. **(B)** HuH-7^{R} cells were incubated with increasing doses of sorafenib for 12 h. The changes of p-ERK and p-YB-1 were examined. Intensity was quantified using densitometry and normalized to that of GAPDH. Blots are representatives of three separate experiments.

Figure S4. Screening of different hepatoma cell lines for sorafenib sensitivity and YB-1 expression. Immunoblotting was performed to assess the protein expression of phosphorylated

YB-1 and YB-1 after long-term sorafenib treatment of HCC cell lines (HuH-7, HuH-7^R, Hep3B, Hep3B^{R,} PLC-5, PLC-5^R, Sk-Hep-1 and Sk-Hep-1^R) using GAPDH as a loading control, upper. Relative changes of the expression levels were quantified using GAPDH as normalization control, lower panel (ns, not significant; *p< 0.05; **p< 0.01).

Figure S5. YB-1 mediates migration and invasion of PLC-5^R cells. (A) PLC-5^R cells were infected with lentiviruses containing shYB-1 (#1, #2) or shCtrl, and after 48 h, lysed and analyzed via immunoblotting with the indicated antibodies. **(B)** Proliferation of PLC-5^R cells treated with shCtrl, shYB-1 and sorafenib determined at the indicated time-points with the MTT assay. Plots depict cumulative cell absorbance versus days in culture. **(C)** Wound-healing assays of YB-1-depleted PLC-5^R cells. The micrographs show cells migrating into the gap 0 h and 24 h after removal of the insert. **(D)** Transwell invasion assay of YB-1-depleted PLC-5^R cells. Cells in the central field of each insert were visualized via light microscopy and quantified. **(E)** Sphere formation assays of YB-1 knockdown PLC-5^R cells. Image of the spheres formed were captured on day 9 and sphere diameter measured. Data are quantified and presented as means ± SD. All results are representative of at least three independent biological replicates (Scale bars = 50 μm). *P* values were determined using the *t* test (ns, not significant; *p< 0.05; **p< 0.01; shCtrl, control shRNA; shYB-1, shRNA against YB-1).

Figure S1

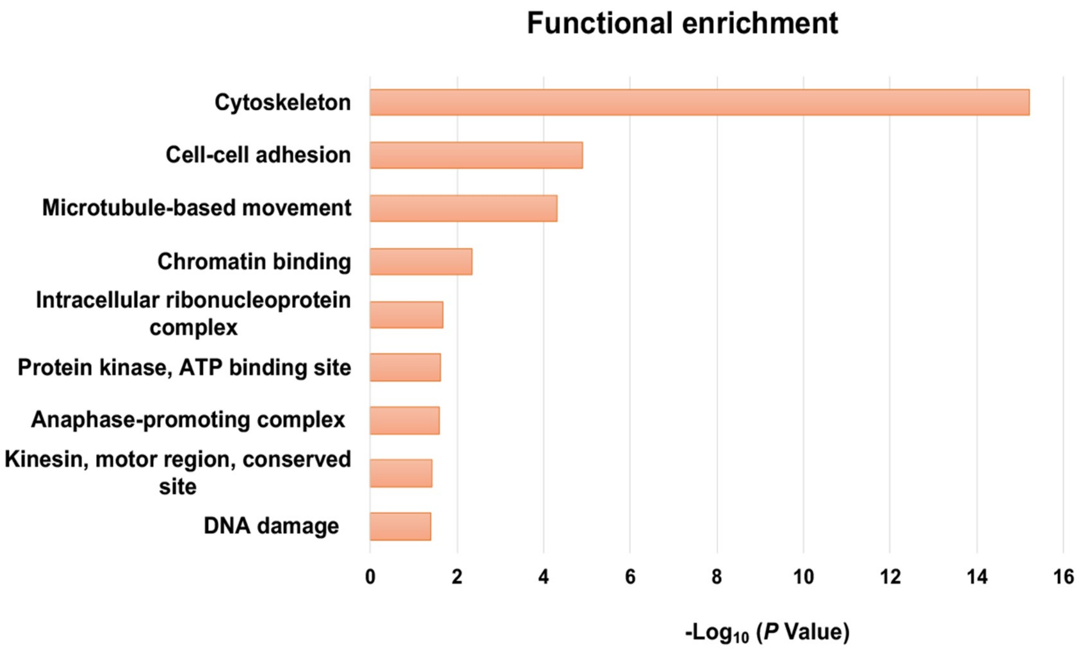
Functional enrichment
Cytoskeleton
Cell-cell adhesion
Microtubule-based movement
Chromatin binding
Intracellular ribonucleoprotein complex
Protein kinase, ATP binding site
Anaphase-promoting complex
Kinesin, motor region, conserved site
DNA damage
0
2
4
6
8
10
12
14
16
-Log10 (P Value)

Figure S2

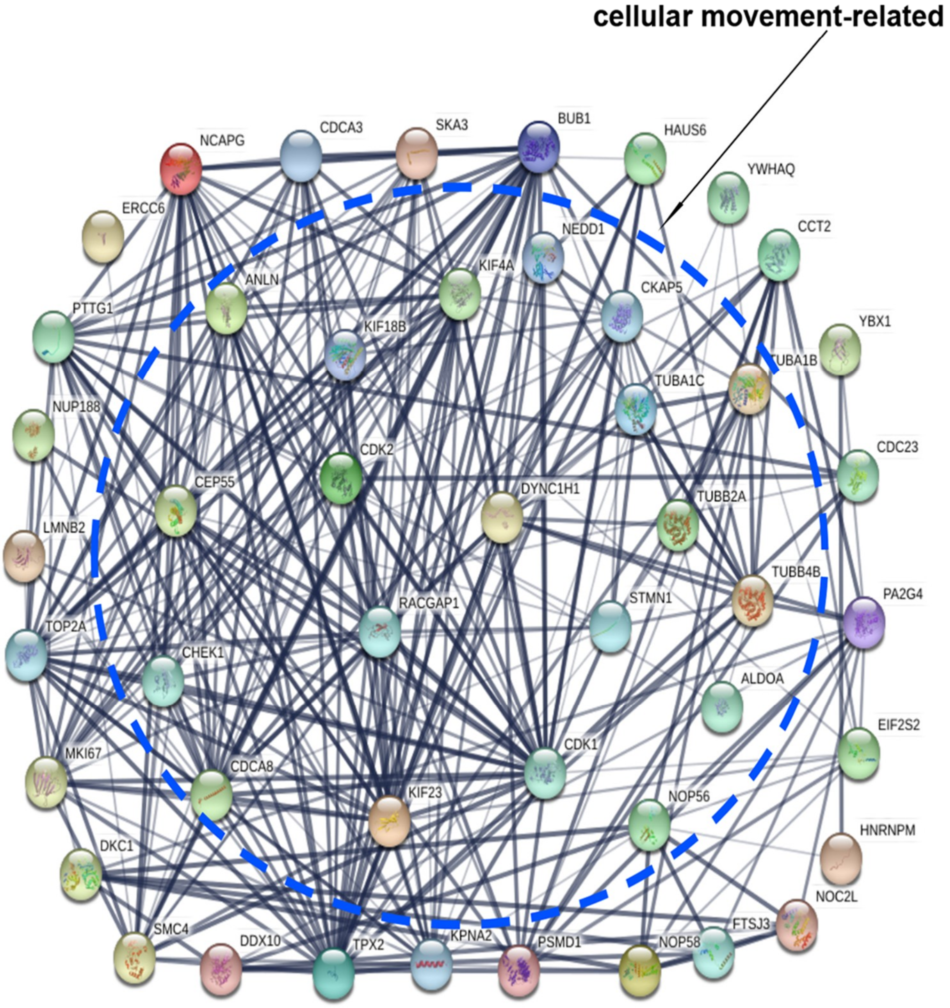
cellular movement-related
NCAPG
CDCA3
SKA3
BUB1
HAUS6
YWHAQ
ERCC6
NEDD1
CCT2
ANLN
KIF4A
CKAP5
PTTG1
YBX1
KIF18B
TUBA1B
TUBA1C
NUP188
CDK2
CDC23
CEP55
DYNC1H1
TUBB2A
LMNB2
TUBB4B
PA2G4
STMN1
RACGAP1
TOP2A
CHEK1
ALDOA
EIF2S2
CDK1
MKI67
CDCA8
KIF23
NOP56
HNRNPM
DKC1
NOC2L
SMC4
DDX10
TPX2
KPNA2
PSMD1
NOP58
FTSJ3

Figure S3

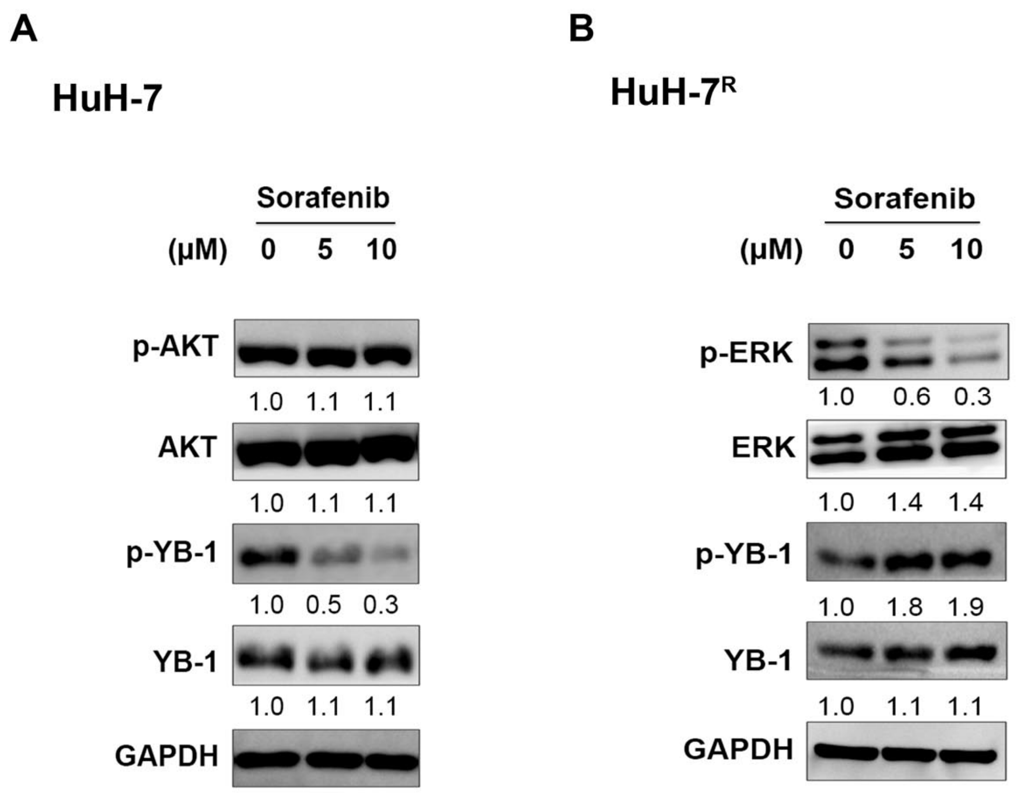
A
HuH-7
Sorafenib
(μM) 0 5 10
p-AKT
1.0 1.1 1.1
AKT
1.0 1.1 1.1
p-YB-1
1.0 0.5 0.3
YB-1
1.0 1.1 1.1
GAPDH
B
HuH-7R
Sorafenib
(μM) 0 5 10
p-ERK
1.0 0.6 0.3
ERK
1.0 1.4 1.4
p-YB-1
1.0 1.8 1.9
YB-1
1.0 1.1 1.1
GAPDH

Figure S4

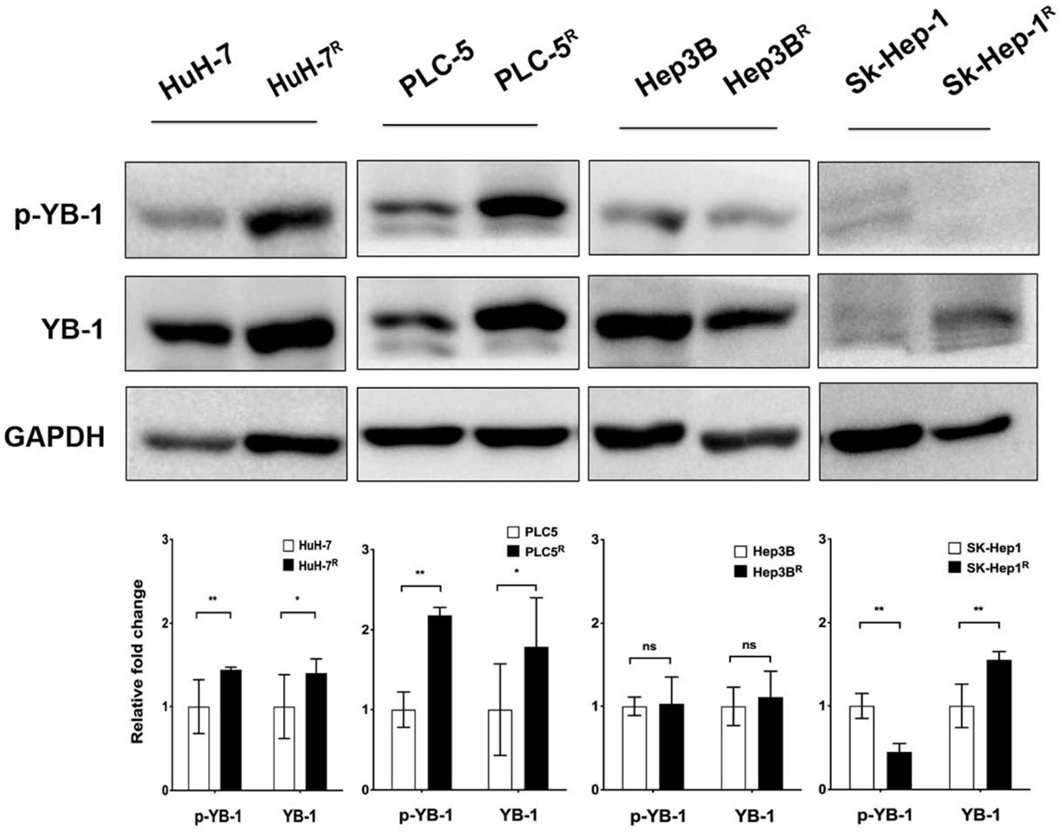

HuH-7
HuH-7R
PLC-5
PLC-5R
Hep3B
Hep3BR
Sk-Hep-1
Sk-Hep-1R
p-YB-1
YB-1
GAPDH
Relative fold change
HuH-7
HuH-7R
PLC5
PLC5R
Hep3B
Hep3BR
SK-Hep1
SK-Hep1R
**
*
ns
p-YB-1
YB-1

Figure S5

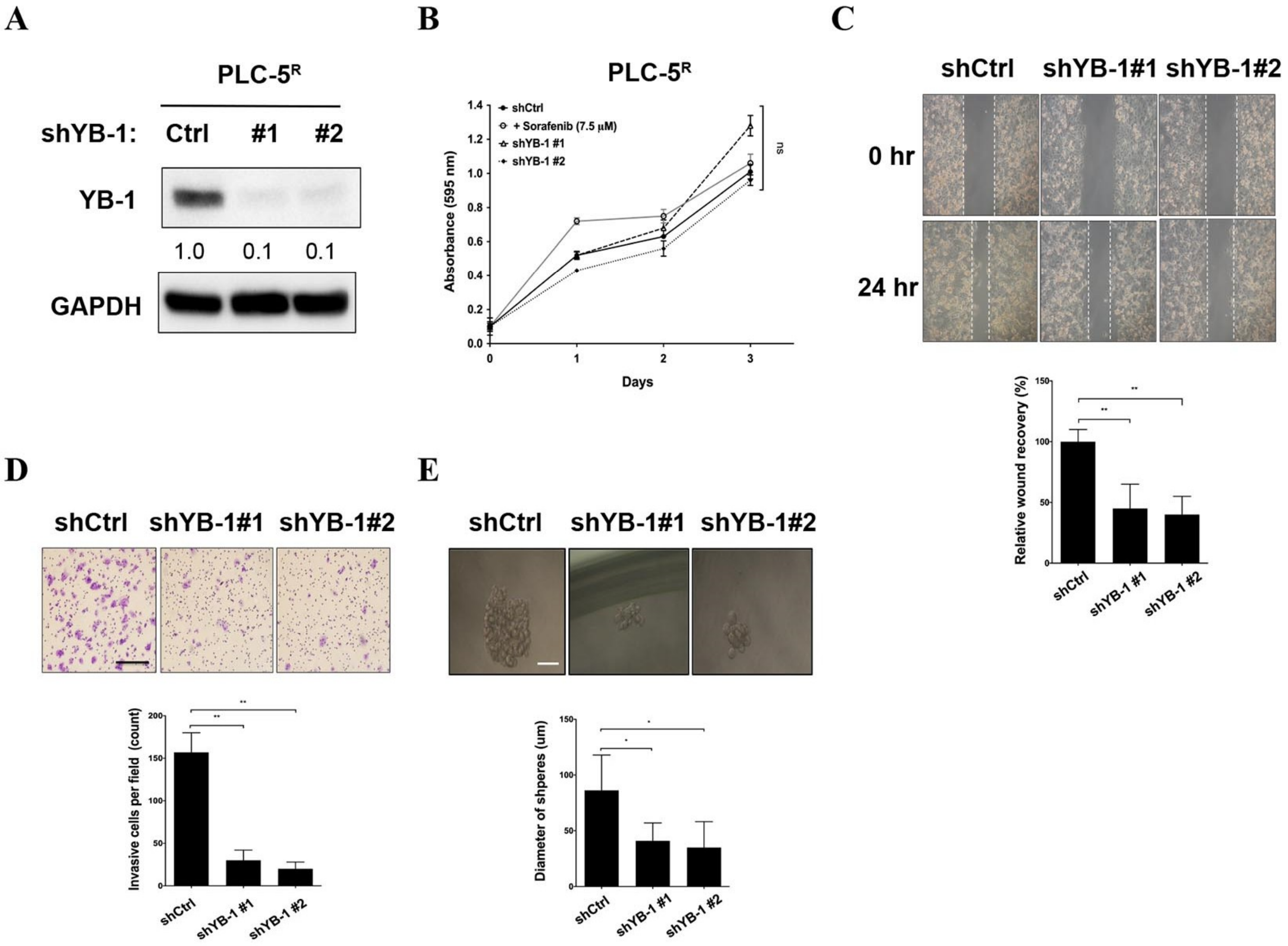
A
PLC-5R
shYB-1: Ctrl #1 #2
YB-1
1.0 0.1 0.1
GAPDH
B
PLC-5R
shCtrl
+ Sorafenib (7.5 μM)
shYB-1 #1
shYB-1 #2
ns
Absorbance (595 nm)
Days
C
shCtrl shYB-1#1 shYB-1#2
0 hr
24 hr
Relative wound recovery (%)
shCtrl
shYB-1 #1
shYB-1 #2
D
shCtrl shYB-1#1 shYB-1#2
Invasive cells per field (count)
shCtrl
shYB-1 #1
shYB-1 #2
E
shCtrl shYB-1#1 shYB-1#2
Diameter of shperes (um)
shCtrl
shYB-1 #1
shYB-1 #2
